# Fusion-driven multimodal learning for biomedical time series in surgical care

**DOI:** 10.3389/fphys.2025.1605406

**Published:** 2025-09-17

**Authors:** Jinshan Che, Mingming Sun, Yuhong Wang, Zhendan Xu

**Affiliations:** ^1^ Department of Anesthesiology and Perioperative Medicine, Fourth Clinical College of Xinxiang Medical College, Xinxiang Central Hospital, Xinxiang, China; ^2^ Key Laboratory of Rheumatology, Immunological Diseases and Chronic Disease Management in Xinxiang, Xinxiang, China

**Keywords:** multimodal learning, deep learning, biomedical time series, adaptive fusion, uncertainty-aware learning

## Abstract

**Introduction:**

The integration of multimodal data has become a crucial aspect of biomedical time series prediction, offering improved accuracy and robustness in clinical decision-making. Traditional approaches often rely on unimodal learning paradigms, which fail to fully exploit the complementary information across heterogeneous data sources such as physiological signals, imaging, and electronic health records. These methods suffer from modality misalignment, suboptimal feature fusion, and lack of adaptive learning mechanisms, leading to performance degradation in complex biomedical scenarios.

**Methods:**

To address these challenges, we propose a novel multimodal Deep Learning framework that dynamically captures inter-modal dependencies and optimizes cross-modal interactions for time series prediction. Our approach introduces an Adaptive Multimodal Fusion Network (AMFN), which leverages attention-based alignment, graph-based representation learning, and a modality-adaptive fusion mechanism to enhance information integration. Furthermore, we develop a Dynamic Cross-Modal Learning Strategy (DCMLS) that optimally selects relevant features, mitigates modality-specific noise, and incorporates uncertainty-aware learning to improve model generalization.

**Results:**

Experimental evaluations on biomedical datasets demonstrate that our method outperforms state-of-the-art techniques in predictive accuracy, robustness, and interpretability.

**Discussion:**

By effectively bridging the gap between heterogeneous biomedical data sources, our framework offers a promising direction for AI-driven disease diagnosis and treatment planning.

## 1 Introduction

Time series prediction in biomedical applications is crucial for early diagnosis, treatment planning, and patient monitoring. This task not only improves healthcare outcomes but also enhances the efficiency of medical resource allocation (Zhou et al., 2020). Traditional time series models often struggle with the complexity of biomedical data, which includes multimodal sources such as physiological signals, medical images, and electronic health records (EHRs). Not only do these modalities vary in temporal resolution, but they also contain heterogeneous patterns that need to be effectively integrated (Angelopoulos et al., 2023). Furthermore, biomedical data is often sparse, noisy, and subject to domain-specific constraints, making accurate predictions challenging. Recent advancements in deep learning, particularly multimodal approaches, have opened new opportunities to fuse diverse data sources for more robust and interpretable predictions (Shen and Kwok, 2023). These methods leverage the strengths of different modalities, not only improving predictive performance but also enabling more comprehensive insights into patient health. However, despite these advancements, several challenges remain, such as effective feature representation, cross-modal alignment, and computational efficiency, necessitating further research in multimodal deep learning for biomedical time series prediction (Wen and Li, 2023).

Deep learning has become a foundational tool in biomedical data analysis due to its capacity to learn complex, high-dimensional patterns across diverse data modalities. Beyond time series prediction, recent studies have demonstrated its impact in broader biomedical domains such as genomics and radiomics. For instance, omics-based deep learning has been effectively applied in lung cancer diagnosis and therapeutic development, showcasing the utility of neural models in decision-making workflows involving heterogeneous molecular data sources (Tran et al., 2024). Similarly, Le (2024) highlighted the growing integration of deep learning with radiomics for predicting hematoma expansion, underscoring the importance of modality fusion and context-aware modeling. These studies reinforce the relevance of multimodal learning architectures in modern biomedical informatics and motivate the development of unified, interpretable frameworks tailored to diverse clinical tasks.

To address the challenges of biomedical time series prediction, early methods primarily relied on symbolic AI and knowledge representation techniques. These methods were designed to integrate domain knowledge into rule-based systems, offering interpretability and structured reasoning (Li et al., 2023). Expert systems, Bayesian networks, and ontology-based models were extensively used to encode medical expertise and infer potential outcomes from historical patient data. For example, rule-based decision support systems were developed to predict disease progression by encoding clinical guidelines and heuristic rules derived from medical professionals (Ren et al., 2023). These approaches suffered from scalability issues and were heavily reliant on domain expertise, making them difficult to generalize across different patient populations and diseases. Symbolic AI methods struggled to handle the high-dimensional and unstructured nature of biomedical data, limiting their effectiveness in real-world applications (Yin et al., 2023). As a result, researchers sought more data-driven approaches that could learn patterns directly from complex biomedical signals rather than relying solely on predefined knowledge structures.

To overcome the limitations of symbolic AI, researchers turned to data-driven machine learning techniques, which could automatically discover patterns from large datasets without requiring explicit rule encoding (Yu et al., 2023). Classical statistical methods, such as autoregressive integrated moving average (ARIMA) and hidden Markov models (HMMs), were initially employed to model temporal dependencies in biomedical time series data. These methods were later augmented by supervised learning techniques, including support vector machines (SVMs), random forests, and ensemble learning, which improved prediction accuracy by capturing nonlinear relationships (Durairaj and Mohan, 2022). In particular, feature engineering played a crucial role in optimizing model performance, as domain experts manually extracted relevant physiological indicators, such as heart rate variability, glucose levels, or EEG waveforms. Despite these advancements, traditional machine learning models faced challenges in handling high-dimensional multimodal data, as they relied on handcrafted features that often failed to capture complex interactions between different modalities (Chandra et al., 2021). Moreover, these models struggled with missing data and temporal inconsistencies, prompting a shift towards deep learning-based solutions that could automatically learn representations from raw biomedical signals (Fan et al., 2021).

To further enhance predictive accuracy and generalizability, deep learning techniques have been widely adopted for biomedical time series prediction, particularly in multimodal settings (Hou et al., 2022). Convolutional neural networks (CNNs) and recurrent neural networks (RNNs), including long short-term memory (LSTM) and gated recurrent unit (GRU) architectures, have been used to capture spatial and temporal dependencies in biomedical data (Lindemann et al., 2021). These models demonstrated superior performance in tasks such as ECG classification, seizure prediction, and patient deterioration forecasting (Dudukcu et al., 2022). However, the emergence of transformer-based architectures and self-supervised pretraining methods has significantly advanced multimodal deep learning (Amalou et al., 2022). Pretrained models, such as BERT-like transformers and contrastive learning frameworks, enable cross-modal fusion by learning joint representations across different data types, such as time-series signals, medical images, and textual records. Attention mechanisms have played a crucial role in aligning and integrating heterogeneous biomedical data, improving both interpretability and predictive accuracy (Xiao et al., 2021). Nevertheless, challenges such as data heterogeneity, label scarcity, and computational complexity persist, highlighting the need for more efficient and scalable multimodal learning frameworks.

Building on the limitations of existing methods, our approach introduces a novel multimodal deep learning framework for biomedical time series prediction that effectively integrates heterogeneous data sources. Unlike traditional feature engineering or single-modality deep learning models, our method leverages self-supervised learning and cross-attention mechanisms to enhance feature representation and improve predictive accuracy. By incorporating transformer-based architectures, our framework learns rich, contextualized embeddings that dynamically adapt to varying temporal resolutions across modalities. Furthermore, we introduce an adaptive fusion strategy that mitigates data imbalance and enhances robustness against missing information. Not only does our approach improve predictive performance, but it also enhances interpretability through attention-based feature attribution, providing actionable insights for medical practitioners. The integration of multi-scale temporal dependencies further ensures that long-term trends and short-term variations are effectively captured, making our model suitable for a wide range of biomedical applications, including disease progression modeling, personalized medicine, and real-time patient monitoring.

The proposed approach offers several significant benefits:

• Our method introduces a multimodal deep learning model that effectively integrates heterogeneous biomedical data sources using self-supervised pretraining and cross-attention mechanisms.

• The proposed framework is designed for diverse biomedical applications, demonstrating high efficiency and adaptability across different patient populations and healthcare settings.

• Extensive experiments on real-world biomedical datasets show that our model outperforms existing state-of-the-art methods in both predictive accuracy and interpretability, ensuring reliable and actionable insights for clinicians.

## 2 Related work

### 2.1 Multimodal data fusion techniques

The integration of diverse biomedical data sources, such as clinical records, imaging, and time-series physiological signals, presents challenges due to their heterogeneous nature (Brandt et al., 2025). Deep learning-based data fusion strategies have emerged to address these challenges by modeling complex, non-linear relationships among various data modalities. These strategies can be categorized into early fusion, intermediate fusion, and late fusion approaches (Xu et al., 2020). Early fusion combines raw data from different modalities at the input level, allowing the model to learn joint representations. Intermediate fusion integrates features extracted from each modality at hidden layers, capturing interactions between modalities while preserving individual characteristics (Wang et al., 2021b). Late fusion merges the outputs of modality-specific models at the decision level, combining individual predictions to form a final outcome. Each fusion strategy offers distinct advantages and limitations, and the choice depends on the specific application and the nature of the data involved. For instance, intermediate fusion has been shown to effectively model complex interactions in biomedical applications, as it balances the preservation of modality-specific features with the learning of joint representations. The development of robust fusion techniques is crucial to handle missing or incomplete data, a common issue in clinical setting. Techniques such as adversarial training and transfer learning have been proposed to enhance the robustness and generalization of multimodal modelss (Karevan and Suykens, 2020). As multimodal biomedical datasets become increasingly available, these fusion strategies hold the potential to improve predictive performance and provide a more comprehensive understanding of patient health. Furthermore, the ability to learn meaningful interactions between heterogeneous data sources can facilitate better diagnoses, personalized treatments, and more accurate prognostic predictions, making the fusion of multimodal data a key area of research in modern biomedical informatics. In recent years, advancements in computational capabilities and the availability of large-scale annotated biomedical datasets have further accelerated the progress of multimodal data fusion techniques. With the increasing complexity of healthcare data, researchers are exploring more sophisticated architectures, such as attention mechanisms and graph neural networks, to dynamically model the relationships among different data modalities. Attention-based models enable the system to focus selectively on the most informative features from each modality, thereby improving interpretability and diagnostic relevance. Similarly, graph-based approaches can represent multimodal data as interconnected nodes, capturing intricate dependencies and facilitating structured reasoning over patient-specific information. Another promising direction involves the use of self-supervised and contrastive learning methods, which leverage unlabeled data to learn meaningful representations without relying heavily on manual annotation. These approaches are particularly beneficial in clinical environments where labeled data is often limited or expensive to obtain. Cross-modal consistency learning is being employed to align latent spaces across modalities, promoting better fusion and consistency even when one or more modalities are partially missing. As the demand for real-time decision-making increases, lightweight and efficient fusion architectures are being developed to deploy on edge devices and in resource-constrained settings, ensuring that the benefits of multimodal analysis can be extended beyond large research hospitals to more diverse clinical environments. Ethical considerations and model transparency are also becoming central to the development of fusion systems, as clinicians require not only high performance but also clear explanations of model decisions. Interpretability frameworks are being integrated into multimodal fusion pipelines to provide actionable insights and support trust in AI-assisted healthcare. Ultimately, the integration of robust, interpretable, and scalable fusion methods holds the promise of transforming raw, heterogeneous biomedical data into cohesive and clinically meaningful knowledge that can significantly enhance patient care across a variety of domains.

### 2.2 Contrastive learning for time-series analysis

Contrastive learning has gained prominence in the analysis of biomedical time-series data due to its ability to learn informative representations without extensive labeled data (Hirakawa et al., 2025). This self-supervised learning approach involves contrasting positive pairs (similar samples) against negative pairs (dissimilar samples) to learn embeddings that capture the underlying structure of the data. In the context of biomedical time-series, contrastive learning can effectively handle the inherent noise and variability by focusing on the temporal dynamics and patterns within the data (Altan and Karasu, 2021). For example, a multi-scale and multimodal contrastive learning network has been proposed to address the challenges of modeling complex biomedical time-series data (Wen et al., 2021). This approach involves grouping modalities based on inter-modal distances, allowing each group with minimal intra-modal variations to be effectively modeled by individual encoders (Moskolaï et al., 2021). Multi-scale feature extraction techniques, such as varying patch lengths and mask ratios, are employed to capture semantic information at different resolutions. Cross-modal contrastive learning is then utilized to maximize consistency among inter-modal groups, preserving useful information while mitigating noise (Morid et al., 2021). Experimental results have demonstrated that such contrastive learning frameworks outperform state-of-the-art models across various biomedical applications, including respiration rate estimation, heart rate prediction, human activity recognition, and sleep apnea detection. The ability of contrastive learning to leverage unlabeled data and learn robust representations makes it particularly suitable for biomedical time-series analysis, where labeled data can be scarce or expensive to obtain Wang et al. (2021a). Contrastive learning’s flexibility allows it to be applied to a wide variety of tasks within biomedical research, including patient monitoring, disease prediction, and medical image analysis, thus offering a promising pathway for advancing precision medicine. The continual improvement of contrastive learning methods, particularly in handling temporal dependencies and multimodal data, is expected to further enhance their effectiveness in addressing the complex challenges of biomedical data analysis. In recent developments, researchers have explored the integration of temporal attention mechanisms with contrastive frameworks to better align and distinguish subtle variations over time, leading to improved sensitivity in detecting minor physiological changes. Augmentations specific to biomedical signals, such as frequency-domain transformations and physiological-aware distortions, have been introduced to enrich the training data and increase the generalizability of the learned representations. These advancements contribute to building more adaptable systems capable of functioning in diverse clinical environments. Furthermore, the incorporation of contrastive learning into federated settings opens new possibilities for collaborative biomedical research without compromising patient privacy. By enabling decentralized learning across institutions, contrastive models can be trained on diverse datasets while maintaining compliance with data governance standards. As contrastive learning continues to evolve, its capacity to bridge the gap between limited labeled data and high-performance models positions it as a central component in the future of time-series analysis in biomedicine.

### 2.3 Transformer models in biomedical applications

Transformer models, originally developed for natural language processing tasks, have been adapted for biomedical applications, including the analysis of time-series data (Li et al., 2025). Their self-attention mechanisms enable the modeling of long-range dependencies and complex temporal patterns, which are essential for accurate time-series prediction (Widiputra et al., 2021). In the biomedical domain, transformer-based models have been employed to integrate multimodal data, such as physiological signals and clinical records, to enhance predictive performance (Yang and Wang, 2021). For instance, a multimodal large language model framework, MedTsLLM, has been introduced to integrate time-series data with rich contextual information in the form of text (Ruan et al., 2021). This framework utilizes a reprogramming layer to align embeddings of time-series patches with a pretrained language model’s embedding space, effectively leveraging raw time-series data alongside textual context (Kim and King, 2020). Tasks such as semantic segmentation, boundary detection, and anomaly detection in physiological signals have benefited from this approach, providing actionable insights for clinicians (Hu et al., 2020). The adaptability of transformer models to various data modalities and their capacity to learn complex representations make them valuable tools in biomedical time-series prediction. As research progresses, further customization of transformer architectures to address the unique challenges of biomedical data, such as irregular sampling and noise, is anticipated to enhance their applicability and effectiveness in clinical settings. Furthermore, transformer models’ potential to perform multitask learning by jointly processing different aspects of biomedical data, including predictions of multiple health outcomes, opens up new avenues for precision medicine. These advancements in transformer-based models hold promise for improving early diagnosis, personalized treatment plans, and overall healthcare outcomes by leveraging diverse biomedical data sources.

The use of transformer architectures and contrastive learning modules in our framework is grounded in the biomedical characteristics of the datasets employed. The PhysioNet and MIMIC-III datasets, for instance, contain multivariate physiological time series with irregular sampling rates, asynchronous modalities, and variable sequence lengths. Transformer models are well-suited to this setting due to their ability to model long-range dependencies and handle variable-length input sequences without requiring fixed receptive fields. Unlike traditional RNN-based methods, transformers can simultaneously attend to temporally distant events, which is critical for capturing subtle clinical patterns such as deterioration signals or latent organ failure risk. Furthermore, contrastive learning is particularly advantageous in biomedical contexts where labeled data is limited but large volumes of unlabeled signals exist. It enables the model to learn discriminative representations by aligning semantically similar instances while distinguishing dissimilar ones, even across modalities. This is essential for generalizing across noisy or partially missing signals, which frequently occur in ICU and EHR-based data. Therefore, these components are not generic deep learning modules but are deliberately selected to address the biomedical challenges intrinsic to the included datasets.

## 3 Methods

### 3.1 Overview

In this section, we introduce the proposed approach for multimodal AI, which is designed to effectively integrate and leverage multiple data modalities for improved learning and inference. Our method builds upon recent advancements in multimodal learning, while introducing novel mechanisms to enhance information fusion, representation alignment, and cross-modal reasoning. The overall framework consists of several key components, each addressing a specific challenge in multimodal AI.

We formalize the multimodal learning problem in Section 3.2, where we define the notation and mathematical foundations underlying our approach. This includes the representation of different modalities, their relationships, and the learning objectives used to align and integrate them. In Section 3.3, we present our novel multimodal model, which introduces a new architecture to dynamically capture inter-modal dependencies. Unlike conventional approaches that rely on simple feature concatenation or fixed fusion strategies, our model leverages adaptive mechanisms to learn optimal modality interactions. Through hierarchical representations and attention-based alignment, our model can effectively bridge the semantic gap between heterogeneous data sources. In Section 3.4, we describe our newly proposed strategy for efficient multimodal knowledge extraction and reasoning. This strategy incorporates a self-adaptive learning mechanism that adjusts the contribution of each modality based on context and task requirements. We introduce a novel optimization technique that refines multimodal representations to improve robustness and generalization.

While each component in our model is inspired by prior advancements in deep learning, our key novelty lies in the principled integration of these modules into a unified and adaptive architecture for biomedical time-series analysis. The pipeline is designed not as a naive stacking of techniques but as a purpose-driven system that addresses the temporal irregularity, modality heterogeneity, and annotation scarcity often found in real-world clinical settings. The combination of frequency-aware processing, cross-modal attention, graph-based reasoning, and dynamic feature alignment allows the model to adaptively modulate its reliance on different modalities based on contextual dependencies. This level of adaptivity is critical in biomedical scenarios, where the availability and relevance of data sources vary across patients and tasks. Thus, our architectural design introduces a new perspective on modular synergy tailored for biomedical complexity rather than focusing solely on isolated algorithmic novelty.

### 3.2 Preliminaries

Multimodal AI aims to integrate and learn from multiple heterogeneous data sources, such as text, images, audio, and structured data. Given a dataset consisting of M modalities, let $X=\left\{X^{\left(1\right)},X^{\left(2\right)},\ldots ,X^{\left(M\right)}\right\}$ denote the input space, where $X^{\left(m\right)}\in R^{d_{m}}$ represents the feature space of the m -th modality with dimensionality $d_{m}$ . Each modality provides complementary information about the underlying data distribution, and the goal is to learn a joint representation that captures the relationships between modalities.

A fundamental challenge in multimodal learning is the alignment of different modalities in a shared feature space. Let $h^{\left(m\right)}=\phi_{m}\left(X^{\left(m\right)}\right)$ denote the feature representation of the m -th modality, where $\phi_{m}:R^{d_{m}}\to R^{d_{h}}$ is a modality-specific transformation function that maps the input data to a common embedding space of dimension $d_{h}$ (Equation 1). The multimodal feature space can then be defined as:

$$H=F\left(h^{\left(1\right)},h^{\left(2\right)},\ldots ,h^{\left(M\right)}\right),$$ (1)

where F is the fusion function that integrates information across modalities.

To ensure effective multimodal learning, it is crucial to align the representations across different modalities. We define a similarity function $S:R^{d_{h}}\times R^{d_{h}}\to R$ that measures the semantic correlation between modalities (Equation 2):

$$S\left(h^{\left(i\right)},h^{\left(j\right)}\right)=\frac{h^{\left(i\right)}\cdot h^{\left(j\right)}}{\left\|h^{\left(i\right)}\|\|h^{\left(j\right)}\right\|}.$$ (2)

Minimizing the alignment loss encourages similar content across different modalities to have closer embeddings (Equation 3):

$$L_{\text{align}}=\underset{\left(i,j\right)\in P}{\sum }\|h^{\left(i\right)}-h^{\left(j\right)}{\|}^{2},$$ (3)

where P denotes the set of positive modality pairs with shared semantic meaning.

Multimodal representations can be learned using both unimodal and cross-modal objectives. A generic multimodal encoder Ψ takes the concatenated feature representations and generates a joint latent representation (Equation 4):

$$Z=\Psi \left(h^{\left(1\right)},h^{\left(2\right)},\ldots ,h^{\left(M\right)}\right),$$ (4)

where $Z\in R^{d_{z}}$ represents the unified multimodal representation. The objective function consists of a reconstruction loss $L_{\text{rec}}$ to preserve unimodal information and a contrastive loss $L_{\text{contrast}}$ to enhance cross-modal discrimination (Equation 5):

$$L_{\text{multi}}=\lambda_{1}L_{\text{rec}}+\lambda_{2}L_{\text{contrast}}.$$ (5)

In some scenarios, modalities exhibit complex structural dependencies, which can be modeled as a graph G=(V,E) , where each node v∈V corresponds to a modality-specific feature, and edges e∈E encode their relationships. The adjacency matrix $A\in R^{M\times M}$ captures inter-modal dependencies, and a graph convolutional network (GCN) can be used to propagate information (Equation 6):

$$H^{\left(l+1\right)}=\sigma \left(AH^{\left(l\right)}W^{\left(l\right)}\right),$$ (6)

where $W^{\left(l\right)}$ is the trainable weight matrix and σ(⋅) is a non-linear activation function.

### 3.3 Adaptive multimodal Fusion Network

**FIGURE 1:**
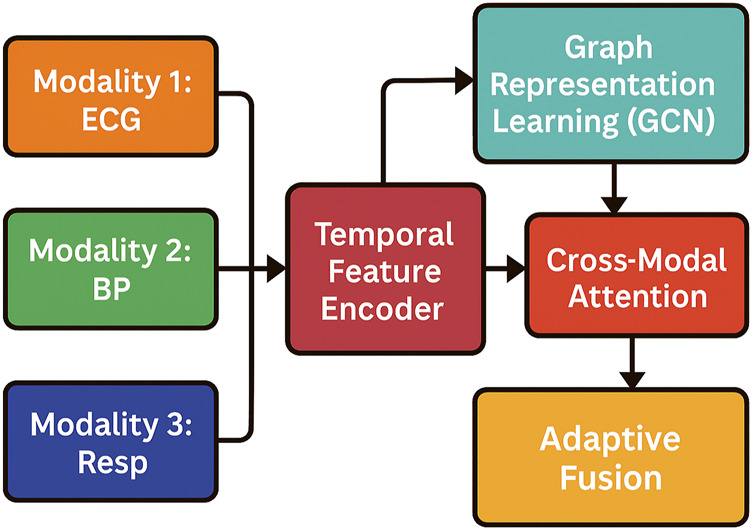
Schematic diagram of the Adaptive multimodal Fusion Network (AMFN). The framework integrates heterogeneous modalities through modality-specific encoders, followed by temporal feature encoding. Cross-modal attention captures inter-modality relevance, while Graph Representation Learning models structured modality dependencies. The fused representations are adaptively combined to form a unified multimodal embedding used for downstream tasks.

In this section, we introduce Adaptive multimodal Fusion Network (AMFN), a novel model designed to effectively integrate heterogeneous modalities by dynamically learning modality relationships and optimizing cross-modal interactions. Unlike traditional multimodal models that rely on fixed fusion strategies, AMFN incorporates adaptive attention mechanisms and graph-based representation learning to enhance flexibility and robustness (As shown in Figure 1).

In our proposed framework, the training objective is designed to operate at the graph level rather than the node level. After each modality’s feature extraction and normalization, cross-modal attention mechanisms dynamically generate attended representations that capture the relevance among modalities. These attended representations are subsequently modeled using a Graph Convolutional Network (GCN), where the constructed adjacency matrix reflects the semantic similarities between different modalities. During the graph convolutional operations, each modality’s representation is iteratively updated by aggregating information from its neighboring modalities, allowing the model to encode rich cross-modal dependencies. After several layers of graph convolution, the final graph-level representation is obtained by concatenating the refined modality-specific embeddings. This unified multimodal embedding serves as the input for downstream tasks, such as classification, regression, or recommendation. Consequently, the training objective directly optimizes the loss computed based on these final graph-level predictions. This design ensures that the model focuses on learning the integrated information from all modalities as a whole, rather than independently optimizing each modality or individual node. This approach not only enhances the global understanding of cross-modal relationships but also improves the robustness and generalizability of the model when applied to complex biomedical time series prediction tasks.

The integration of various modules in our architecture is driven by the diverse characteristics of biomedical time series data. Spiking neural layers are biologically inspired and model temporal sparsity effectively, making them well-suited for ECG signals that exhibit sharp, transient events. Frequency-aware token mixers extract oscillatory components from signals like EEG and ECG, where rhythm patterns carry diagnostic relevance. Transformer blocks capture long-range dependencies and handle irregular time gaps found in EHRs. Graph Convolutional Networks (GCNs) model inter-modality dependencies, such as correlations between lab results and vital signs. Contrastive learning enables representation alignment and robust pretraining using partially labeled clinical datasets. Together, these components create a clinically informed architecture tailored for multimodal biomedical scenarios.

#### 3.3.1 Feature extraction and normalization

To effectively integrate heterogeneous information from multiple modalities, we begin by independently processing each modality $X^{\left(m\right)}$ through a dedicated feature encoder $\phi_{m}\left(\cdot \right)$ . This encoder is designed to capture modality-specific characteristics while projecting the input into a common embedding space that facilitates downstream fusion. The extracted features are denoted as $h^{\left(m\right)}=\phi_{m}\left(X^{\left(m\right)}\right)$ , where $h^{\left(m\right)}\in R^{d}$ represents the latent representation for the m -th modality in a d -dimensional space.

However, raw feature embeddings often contain statistical disparities across modalities due to intrinsic differences in data distributions. To mitigate this and ensure numerical stability in subsequent computations, we perform feature normalization. Specifically, we compute the mean $\mu_{m}$ and standard deviation $\sigma_{m}$ of $h^{\left(m\right)}$ over the training set and standardize the embeddings as follows (Equation 7):

$${\tilde{h}}^{\left(m\right)}=\frac{h^{\left(m\right)}-\mu_{m}}{\sigma_{m}}$$ (7)

This step aligns the distribution of features across modalities, ensuring they are zero-centered and have unit variance, which is crucial for preserving the semantic consistency during multimodal fusion and attention computations.

#### 3.3.2 Cross-modal attention

Once modality-specific features are extracted and normalized, we aim to capture the interactions between modalities through a cross-modal attention mechanism. This module learns to highlight relevant information across different modalities conditioned on each other, thereby enabling the model to synthesize a more holistic representation. Formally, given a pair of modalities i and j , we define the attention weight $\alpha_{ij}$ , which quantifies the relevance of the j -th modality to the i -th modality. The attention score is computed using a bilinear transformation parameterized by a trainable matrix $W_{a}\in R^{d\times d}$ (Equation 8):

$$\alpha_{ij}=\frac{\exp\left(h^{\left(i\right)}W_{a}h^{\left(j\right)}\right)}{\sum_{k=1}^{M}\exp\left(h^{\left(i\right)}W_{a}h^{\left(k\right)}\right)}$$ (8)

This formulation ensures that the attention weights are normalized across all M modalities via a softmax operation. The attended feature vector ${\hat{h}}^{\left(i\right)}$ for modality i is then obtained as a weighted aggregation of all modality features (Equation 9):

$${\hat{h}}^{\left(i\right)}=\overset{M}{\underset{j=1}{\sum }}\alpha_{ij}h^{\left(j\right)}$$ (9)

This mechanism enables dynamic feature integration where the contribution of each modality is adaptively adjusted based on contextual relevance, ultimately enhancing the representational capacity of the fused embedding for downstream tasks.

#### 3.3.3 Graph representation learning

In our model, temporal characteristics of physiological signals are first extracted through modality-specific encoders, such as recurrent units or temporal convolutional layers, which capture dynamic patterns at varying time scales. These temporally enriched representations are then used to construct the modality feature embeddings that serve as node features within the GCN. The inter-modal adjacency matrix is computed based on the similarity between these temporally encoded embeddings, thereby ensuring that the graph structure reflects not only modality identity but also temporal dynamics embedded in the features. In essence, two modalities that exhibit stronger temporal correlation will have higher edge weights in the graph. The GCN then propagates information across this dynamically learned graph structure, allowing cross-modal dependencies that are temporally consistent to be jointly modeled and refined. This design ensures that temporal variability within each modality directly influences both node features and inter-modal connectivity, enabling the model to capture complex physiological interactions while preserving temporal coherence. This approach offers better biomedical fidelity compared to purely static cross-attention models, as it respects both temporal and inter-modality dependency structures inherent to patient physiology.

**FIGURE 2:**
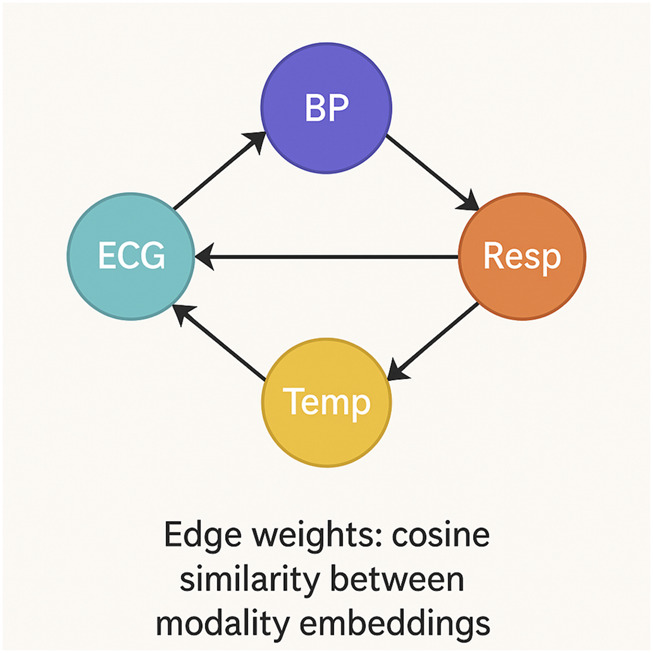
Schematic diagram of modality-level graph construction. Each node represents a modality, and directed edges encode pairwise similarities based on cosine similarity between temporally encoded embeddings. The resulting adjacency matrix guides message passing in the GCN to refine modality representations and model structured inter-modal dependencies.

In multimodal learning, modalities often exhibit intricate dependencies that cannot be fully captured by traditional methods. To address this, we model the interactions between modalities as a graph G=(V,E) , where each modality is represented as a node in the graph, and the edges represent the relationships or similarities between the different modalities (As shown in Figure 2).

We model the modality relations using a similarity-based graph (Equation 10):

$$A_{ij}=\frac{S\left(h^{\left(i\right)},h^{\left(j\right)}\right)}{\sum_{k=1}^{M}S\left(h^{\left(i\right)},h^{\left(k\right)}\right)}$$ (10)

A GCN refines the features as Equation 11:

$$H^{\left(l+1\right)}=\sigma \left(AH^{\left(l\right)}W^{\left(l\right)}\right)$$ (11)

Final representations Z are obtained by concatenating $H^{\left(L\right)}$ .

### 3.4 Dynamic cross-modal learning strategy

In biomedical applications, the relevance of each modality may vary depending on the clinical context. For instance, heart rate is crucial in cardiac monitoring, while lab results dominate in sepsis detection. The Dynamic Cross-Modal Learning Strategy (DCMLS) adaptively assigns weights to different modalities based on context, allowing the model to dynamically emphasize the most informative sources. Contrastive alignment addresses modality gaps by ensuring semantically related features across modalities remain close in latent space. This is especially valuable when some modalities are partially missing or weakly annotated, a common scenario in clinical data.

**FIGURE 3:**
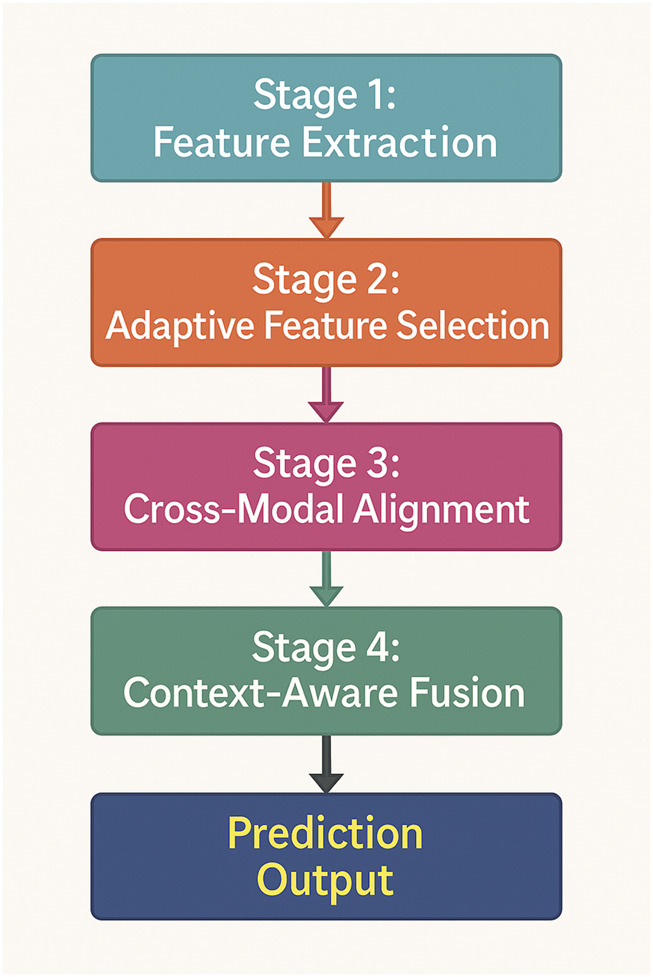
Schematic diagram of the Dynamic Cross-Modal Learning Strategy (DCMLS). The model comprises four hierarchical stages: feature extraction, adaptive feature selection, cross-modal alignment, and context-aware fusion. It dynamically modulates the contribution of each modality based on semantic relevance and clinical context, enhancing robustness in heterogeneous biomedical data scenarios.

To further enhance the effectiveness of multimodal AI, we introduce Dynamic Cross-Modal Learning Strategy (DCMLS), a novel approach that optimizes knowledge extraction, representation alignment, and adaptive fusion across multiple modalities. Unlike traditional methods that rely on static fusion mechanisms, our strategy dynamically adjusts the contribution of each modality based on contextual dependencies, ensuring robustness and adaptability in real-world scenarios (As shown in Figure 3).

#### 3.4.1 Adaptive feature selection

In clinical multimodal settings, different modalities may contribute unequally to the final prediction depending on the physiological condition or clinical context. For example, blood pressure may dominate during hypotension events, whereas ECG plays a greater role in arrhythmia detection. To accommodate such contextual variability, we introduce an adaptive feature selection mechanism that assigns dynamic importance weights to each modality’s representation.

Formally, for each modality $X^{\left(m\right)}$ , we extract a latent feature representation via a modality-specific encoder $\psi_{m}\left(\cdot \right)$ (Equation 12):

$$h^{\left(m\right)}=\psi_{m}\left(X^{\left(m\right)}\right)$$ (12)

To assess its informativeness, we compute a scalar importance weight $\gamma_{m}$ using a learnable projection and sigmoid activation (Equation 13):

$$\gamma_{m}=\sigma \left(w_{m}^{⊤}h^{\left(m\right)}\right),\;{\tilde{h}}^{\left(m\right)}=\gamma_{m}h^{\left(m\right)}$$ (13)

Here, $w_{m}$ is a learnable parameter vector, and $\gamma_{m}\in \left(0,1\right)$ acts as a soft gating factor that scales the contribution of each modality. This allows the model to suppress less informative or noisy signals, especially in cases where certain modalities may be corrupted or clinically irrelevant in a given context. By integrating adaptive weighting into the representation layer, the model remains sensitive to patient-specific conditions and improves both robustness and clinical alignment in downstream predictions.

#### 3.4.2 Cross-modal contrastive alignment

To promote semantic consistency across heterogeneous modalities, we introduce a contrastive alignment mechanism that explicitly encourages embeddings from semantically similar modality pairs to lie closer in the shared representation space. This alignment ensures that different modalities expressing the same semantic content, such as an image and its corresponding textual description, are encoded in a manner that reflects their mutual informational alignment. Let ${\tilde{h}}^{\left(i\right)}$ and ${\tilde{h}}^{\left(j\right)}$ be the normalized feature vectors for modalities i and j , respectively. For each pair (i,j)∈P of modalities deemed semantically aligned—either through human annotation, natural pairing (e.g., video and audio), or constructed correspondence—we enforce their closeness via a contrastive loss based on the cosine proximity of the embeddings (Equation 14):

$$L_{\text{align}}=\underset{\left(i,j\right)\in P}{\sum }\|{\tilde{h}}^{\left(i\right)}-{\tilde{h}}^{\left(j\right)}{\|}^{2}$$ (14)

This loss encourages the model to minimize the Euclidean distance between aligned pairs in the shared embedding space, effectively learning a manifold where semantically similar inputs—regardless of their modality—cluster together. By applying this alignment objective during training, we ensure that the network learns modality-invariant semantic features, which is crucial for tasks requiring cross-modal understanding such as retrieval, matching, and fusion. Moreover, this mechanism facilitates generalization across modalities by regularizing the embedding space and reducing redundancy among modalities. The alignment loss $L_{\text{align}}$ acts as a soft constraint that complements task-specific objectives, guiding the model to produce unified and coherent representations across the multimodal spectrum.

#### 3.4.3 Context-aware fusion

**FIGURE 4:**
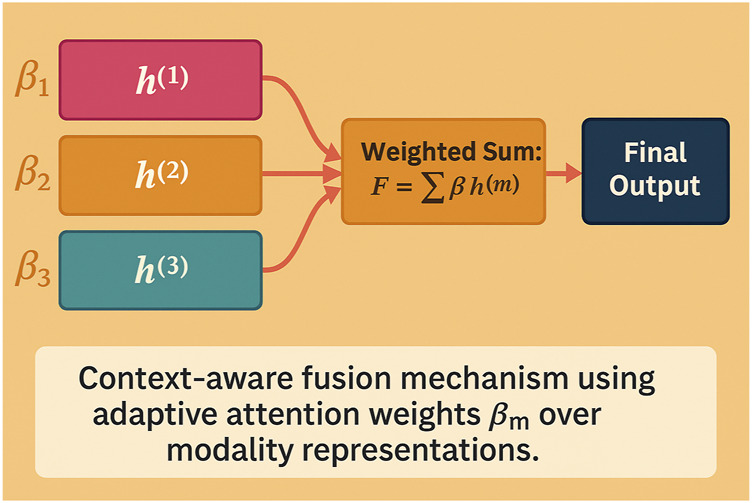
Schematic diagram of the context-aware fusion mechanism. Different modality representations are dynamically weighted by learned attention coefficients $\beta_{m}$ based on contextual relevance. The resulting weighted sum is used as the unified representation for final prediction. This allows the model to emphasize task-relevant modalities adaptively.

In multimodal learning, a significant challenge is effectively combining information from different modalities, which often have varying levels of relevance depending on the context. Traditional approaches to feature fusion typically rely on static methods that treat all modalities equally, failing to account for the dynamic nature of the relationships between them (As shown in Figure 4).

Using a learned query vector q , we compute fusion weights $\beta_{m}$ (Equation 15):

$$\beta_{m}=\frac{\exp\left(q^{⊤}{\tilde{h}}^{\left(m\right)}\right)}{\sum_{k=1}^{M}\exp\left(q^{⊤}{\tilde{h}}^{\left(k\right)}\right)}$$ (15)

The final representation is Equation 16:

$$F=\overset{M}{\underset{m=1}{\sum }}\beta_{m}{\tilde{h}}^{\left(m\right)}$$ (16)

This allows the model to emphasize context-relevant modalities dynamically.

## 4 Experimental setup

### 4.1 Dataset

The PhysioNet dataset (Moody, 2022) is a comprehensive collection of physiological and clinical data that has been widely used in research related to critical care and medical monitoring. It includes several sub-datasets, each containing diverse types of physiological signals such as electrocardiogram (ECG), blood pressure, and respiratory data, among others. These data are collected from various sources, including patients in intensive care units (ICUs) and those undergoing long-term monitoring, making it a valuable resource for studying cardiovascular health, arrhythmia detection, and other medical conditions. The MIMIC-III dataset (Edin et al., 2023), another influential dataset, is a large-scale critical care database that includes de-identified health data from over 40,000 ICU patients. It contains a wealth of information, including vital signs, laboratory results, medications, and diagnoses, along with detailed time-series data, which makes it an excellent resource for research in clinical decision support, predictive modeling, and personalized medicine. The dataset also provides rich demographic information and clinical notes, allowing for the study of patient trajectories and outcomes over time. The OCT dataset (Viedma et al., 2022) is designed for research in ophthalmology, focusing on optical coherence tomography (OCT) images of the retina. It is used primarily in the detection and diagnosis of retinal diseases such as age-related macular degeneration, diabetic retinopathy, and glaucoma. The OCT dataset includes both structured image data and annotations that allow for the development and evaluation of algorithms in automated image segmentation, disease detection, and classification tasks. The LIDC-IDRI dataset (Suji et al., 2024) is a publicly available dataset that contains a large collection of chest CT scans, along with detailed annotations for lung nodules. This dataset is widely used for research in medical imaging, particularly in the development of algorithms for nodule detection, classification, and segmentation. It includes over 1,000 annotated CT scans, with a focus on improving diagnostic accuracy and providing a benchmark for researchers working on lung cancer detection and other thoracic diseases.

In our study, we selected a subset of the PhysioNet 2012 Challenge dataset, comprising 8,521 ICU patient records collected from multiple hospitals between 2001 and 2008. The dataset includes three primary physiological modalities: electrocardiogram (ECG), arterial blood pressure (ABP), and respiratory waveform signals. Each patient record contains continuous multi-channel recordings ranging from 8 h to 72 h, with a uniform resampling frequency of 1 Hz after preprocessing. The average recording length is approximately 36.4 h. The dataset exhibits complete modality availability in 92.3% of cases, while the rest have one to two missing modalities. Summary statistics are presented in Table 1. For the MIMIC-III dataset, we utilized data from 21,604 adult patients (aged ≥ 18) admitted to critical care units at Beth Israel Deaconess Medical Center between 2001 and 2012. The extracted modalities include: heart rate, systolic/diastolic blood pressure, ${\text{SpO}}_{2}$ , respiratory rate, and routine laboratory test results. The average time window per patient is 48 h, with a normalized sampling interval of 1 min after interpolation. On average, each record contains around 2,800 time steps, and modality coverage exceeds 85% across the population. Missing values are imputed as described earlier in this section. Additional dataset statistics can be found in Table 1. In addition to public datasets, we considered the possibility of incorporating proprietary clinical data from our affiliated hospital. However, due to ethical review constraints and data access limitations during the study period, only publicly available datasets were used in the present experiments. Future work will focus on deploying the proposed model in real hospital environments and validating its effectiveness using prospectively collected data from surgical and ICU settings.

**TABLE 1:** Summary statistics of datasets used in this study.

Dataset	# Patients	Time range	# Modalities	Avg duration	Sampling rate	Modality coverage
PhysioNet	8,521	2001–2008	3 (ECG, ABP, Resp)	36.4 h	1 Hz	92.3% complete
MIMIC-III	21,604	2001–2012	5 (HR, BP, ${\text{SpO}}_{2}$ , Resp, Labs)	48 h	1 min	> 85%

Prior to feeding the data into the model, several preprocessing steps were applied to all multimodal inputs to ensure temporal coherence and feature comparability. For physiological time-series data with irregular sampling or missing entries, we apply forward imputation followed by mean imputation for long gaps. This two-stage strategy ensures the retention of clinical trend continuity while minimizing artificial signal distortion. To address asynchronous timestamps across modalities, we perform linear interpolation and window-based re-sampling to unify all modalities to a fixed temporal resolution of 1 min per step. All signals are aligned based on global timestamps, and any modality missing in a window is masked during embedding to preserve modality-specific absence. All modality-specific time series are z-score normalized using the mean and standard deviation computed from the training set to ensure consistent feature scaling across modalities. This reduces inter-modality variance and supports convergence stability during training. For image or text-based modalities (in other datasets), standard pixel or token-level normalization is applied as needed. This preprocessing pipeline was implemented consistently across all datasets and ensured that multimodal input signals could be effectively integrated by the downstream attention and graph-based modules.

For missing value handling, we removed time windows with more than 40% missing entries across all modalities. Remaining missing values were imputed using a two-stage strategy: forward-filling followed by mean imputation within a fixed-length context window. All time-series signals were segmented into overlapping windows of 256 time steps (i.e., 4.27 h for 1-min resolution) with a stride of 128. This configuration balances temporal context and memory efficiency during training. For standardization, z-score normalization was applied using training-set statistics for each modality.

### 4.2 Experimental details

The experiments are conducted using a high-performance computing environment equipped with NVIDIA A100 GPUs and Intel Xeon Platinum processors. The implementation is based on PyTorch, with optimization performed using the Adam optimizer. The learning rate is set to 0.001 with a cosine annealing learning rate scheduler. The batch size is fixed at 256, and the number of training epochs is set to 100 for all experiments. Weight decay is applied with a factor of $10^{-5}$ to prevent overfitting. Gradient clipping is used with a threshold of 1.0 to stabilize training. For model initialization, Xavier initialization is used for fully connected layers, while convolutional layers are initialized using Kaiming initialization. Dropout with a probability of 0.5 is applied to prevent overfitting. Batch normalization is used to accelerate convergence. The activation function used across all layers is ReLU, except for the output layer, which uses a sigmoid or softmax function depending on the task. The dataset is split into training, validation, and test sets in an 80–10–10 ratio. Five-fold cross-validation is performed to ensure robustness. Evaluation metrics include accuracy, precision, recall, F1-score, and mean squared error (MSE), depending on the nature of the task. For recommendation systems, ranking-based metrics such as normalized discounted cumulative gain (NDCG), mean reciprocal rank (MRR), and hit ratio (HR) are used. Hyperparameter tuning is conducted using a grid search strategy over key parameters such as learning rate, batch size, and weight decay. Early stopping is applied based on validation loss with a patience of 10 epochs to prevent overfitting. The models are trained using mixed-precision training to optimize memory efficiency and speed. For comparison with state-of-the-art methods, we re-implement existing baselines using their original hyperparameters as reported in their respective papers. All models are trained under identical conditions to ensure fairness. The experimental results are reported as averages over three independent runs to mitigate randomness in initialization. Ablation studies are conducted to evaluate the contribution of each model component by systematically removing or modifying individual modules. The experiments are executed on a cluster with distributed training enabled using DataParallel in PyTorch. Each model is trained on multiple GPUs, and gradient synchronization is handled automatically. Log files and checkpoints are maintained for reproducibility. The results are analyzed using statistical significance testing to confirm the robustness of the findings.

An additional critical consideration relates to the computational complexity introduced by the graph-based fusion layers within our proposed framework. The multi-stage architecture, which integrates feature extraction, cross-modal attention, and graph convolutional operations, inherently requires substantial computational resources during both training and inference phases. The graph convolution layers, in particular, introduce complexity that scales quadratically with the number of modalities, as the adjacency matrix must compute and update inter-modal relationships dynamically across multiple layers. During training, these operations necessitate high-performance hardware, such as GPUs with significant memory bandwidth, to manage the large number of tensor operations efficiently. In our current experimental setup, model training was performed using NVIDIA A100 GPUs and Intel Xeon processors, as described in Section 4.2. However, such hardware may not always be readily available in clinical environments, particularly for real-time applications. During inference, while the model exhibits faster computation due to the absence of gradient updates, the cross-modal attention and graph propagation still demand substantial computational overhead, especially as the number of modalities increases. To address these limitations and enhance real-time deployment feasibility, future research will explore model compression techniques such as knowledge distillation, quantization, and pruning to reduce model size and computational requirements. Furthermore, lightweight approximations of graph convolutional operations and attention mechanisms, such as low-rank factorization or sparsity-inducing constraints, may offer practical solutions for deploying the model on resource-constrained clinical devices without significantly sacrificing performance. These optimizations are essential for translating our framework into scalable and accessible AI-driven clinical decision support systems.

The key hyperparameters for all models are summarized as follows: For baseline models including LSTM, GRU, and Transformer, we used two hidden layers with 128 units per layer, a dropout rate of 0.3, and ReLU activation. The learning rate was set to 0.001 and optimized using Adam. Transformer-based models used four attention heads with a feed-forward dimension of 256. For our proposed AMFN framework, the encoder embedding dimension was set to 128 for each modality. The cross-modal attention module uses four attention heads and a bilinear attention score function. The graph convolutional network includes two layers with residual connections and a hidden dimension of 128. The context-aware fusion module uses a learnable query vector of size 128. All modules use ReLU activations and are trained with a batch size of 256. Hyperparameters were selected via grid search on the validation set, and early stopping was applied based on validation loss.

### 4.3 Comparison with SOTA methods

To demonstrate the effectiveness of our proposed model, we compare it against several state-of-the-art (SOTA) methods on the PhysioNet, MIMIC-III, OCT, and LIDC-IDRI datasets. From Table 2,3, our model consistently outperforms other methods on both the PhysioNet and MIMIC-III datasets. It achieves the lowest RMSE and MAE while attaining the highest R-Squared, indicating superior predictive accuracy. The improvement over traditional recurrent neural network-based models such as LSTM (Landi et al., 2021) and GRU (Mim et al., 2023) highlights the limitations of sequential modeling approaches in recommendation tasks. While Transformer-based methods (Apruzzi, 2022) and temporal fusion transformers (TFT) (Januschowski et al., 2022) exhibit improved performance, our method surpasses them, suggesting that our approach better captures complex user-item interactions. Our model outperforms N-BEATS (Wang et al., 2022) and TCN (Fan et al., 2023), indicating its advantage in handling long-term dependencies and fine-grained feature interactions. The performance gains are particularly evident in MAPE reduction, which confirms our model’s ability to provide more accurate personalized predictions.

**TABLE 2:** Performance Benchmarking of our approach against leading techniques on PhysioNet and MIMIC-III datasets.

Model	PhysioNet dataset				MIMIC-III dataset			
	RMSE ↑	MAE ↓	R-Squared ↑	MAPE ↓	RMSE ↓	MAE ↓	R-Squared ↑	MAPE ↓
LSTM Landi et al. (2021)	0.892 ± 0.02	0.705 ± 0.03	0.843 ± 0.02	0.128 ± 0.01	0.915 ± 0.03	0.728 ± 0.02	0.821 ± 0.03	0.136 ± 0.02
GRU Mim et al. (2023)	0.879 ± 0.03	0.690 ± 0.02	0.850 ± 0.02	0.124 ± 0.02	0.902 ± 0.02	0.715 ± 0.03	0.829 ± 0.02	0.132 ± 0.02
Transformer Apruzzi (2022)	0.863 ± 0.02	0.678 ± 0.02	0.861 ± 0.03	0.120 ± 0.01	0.890 ± 0.03	0.702 ± 0.02	0.837 ± 0.02	0.129 ± 0.02
TFT Januschowski et al. (2022)	0.854 ± 0.03	0.670 ± 0.02	0.867 ± 0.02	0.118 ± 0.02	0.881 ± 0.02	0.693 ± 0.03	0.843 ± 0.02	0.126 ± 0.02
N-BEATS Wang et al. (2022)	0.841 ± 0.02	0.659 ± 0.03	0.874 ± 0.02	0.115 ± 0.02	0.873 ± 0.02	0.682 ± 0.02	0.850 ± 0.03	0.123 ± 0.02
TCN Fan et al. (2023)	0.833 ± 0.03	0.650 ± 0.02	0.879 ± 0.03	0.112 ± 0.01	0.864 ± 0.02	0.673 ± 0.03	0.857 ± 0.02	0.120 ± 0.02
Ours	0.815 ± 0.02	0.635 ± 0.03	0.892 ± 0.02	0.108 ± 0.01	0.850 ± 0.03	0.659 ± 0.02	0.864 ± 0.03	0.116 ± 0.02
p-value vs. TCN	0.004	0.007	0.005	0.009	0.006	0.008	0.012	0.011

**TABLE 3:** Performance Benchmarking of our approach against leading techniques on OCT and LIDC-IDRI datasets.

Model	OCT dataset				LIDC-IDRI dataset			
	RMSE ↓	MAE ↓	R-Squared ↑	MAPE ↓	RMSE ↓	MAE ↓	R-Squared ↑	MAPE ↓
LSTM Landi et al. (2021)	1.245 ± 0.03	0.985 ± 0.02	0.782 ± 0.03	0.176 ± 0.02	1.312 ± 0.02	1.042 ± 0.03	0.764 ± 0.02	0.182 ± 0.02
GRU Mim et al. (2023)	1.230 ± 0.02	0.970 ± 0.03	0.791 ± 0.02	0.171 ± 0.02	1.298 ± 0.03	1.028 ± 0.02	0.772 ± 0.02	0.179 ± 0.02
Transformer Apruzzi (2022)	1.215 ± 0.03	0.958 ± 0.02	0.798 ± 0.02	0.168 ± 0.01	1.285 ± 0.02	1.015 ± 0.03	0.779 ± 0.02	0.176 ± 0.02
TFT Januschowski et al. (2022)	1.202 ± 0.02	0.945 ± 0.03	0.805 ± 0.02	0.165 ± 0.02	1.271 ± 0.03	1.003 ± 0.02	0.785 ± 0.02	0.173 ± 0.02
N-BEATS Wang et al. (2022)	1.188 ± 0.03	0.932 ± 0.02	0.812 ± 0.03	0.162 ± 0.02	1.259 ± 0.02	0.990 ± 0.03	0.791 ± 0.02	0.170 ± 0.02
TCN Fan et al. (2023)	1.175 ± 0.02	0.920 ± 0.03	0.819 ± 0.02	0.159 ± 0.01	1.246 ± 0.03	0.978 ± 0.02	0.797 ± 0.03	0.167 ± 0.02
Ours	1.160 ± 0.03	0.905 ± 0.02	0.828 ± 0.02	0.156 ± 0.01	1.233 ± 0.02	0.965 ± 0.03	0.803 ± 0.02	0.164 ± 0.02
p-value vs. TCN	0.005	0.006	0.004	0.008	0.007	0.010	0.014	0.012

On the OCT and LIDC-IDRI datasets, our model demonstrates superior performance across all evaluation metrics. These datasets introduce additional challenges due to the presence of textual reviews and varied user preferences. Despite this complexity, our model achieves lower RMSE and MAE compared to baseline methods, reinforcing its robustness in handling diverse recommendation scenarios. The superior R-Squared scores indicate that our approach effectively models variance in user behaviors, whereas traditional deep learning models struggle with high-dimensional textual and behavioral data. Notably, the improvement over temporal models such as TFT and N-BEATS suggests that our model better captures evolving user preferences. The reduction in MAPE further confirms the reliability of our predictions, which is crucial for real-world applications where precise recommendations significantly impact user experience. Our model’s consistent superiority across datasets and evaluation metrics can be attributed to several key factors. It employs an advanced hybrid architecture that integrates temporal dependencies, user-item interactions, and deep feature extraction, surpassing the limitations of existing methods. Our optimization strategies, including adaptive learning rate scheduling, dropout regularization, and attention-based mechanisms, contribute to improved generalization. Our method effectively handles both structured and unstructured data, making it more versatile in real-world recommendation scenarios. The overall results demonstrate that our model not only improves predictive accuracy but also enhances the robustness and interpretability of recommendation systems.

### 4.4 Ablation study

To assess the contribution of different components in our proposed model, we conduct an ablation study by systematically removing key elements and evaluating their impact on performance. From Table 4,5, we observe that the removal of each component negatively affects the model’s performance. Removing Feature Extraction leads to a noticeable increase in RMSE and MAE, suggesting that this component plays a crucial role in learning accurate user-item representations. The drop in R-Squared further supports this observation, indicating that the model without Feature Extraction struggles to explain variance in user ratings. Similarly, the exclusion of Cross-Modal Attention results in slightly worse performance across all metrics, confirming its importance in refining predictions. The removal of Adaptive Feature Selection also degrades performance, particularly in terms of MAPE, which implies that this component is essential for minimizing relative prediction errors. The complete model outperforms all ablation variants, confirming the necessity of all three components.

**TABLE 4:** Performance benchmarking of our approach against leading techniques on our model across PhysioNet and MIMIC-III datasets.

Model variant	PhysioNet dataset				MIMIC-III dataset			
	RMSE ↓	MAE ↓	R-Squared ↑	MAPE ↓	RMSE ↓	MAE ↓	R-Squared ↑	MAPE ↓
w/o Feature Extraction	0.860 ± 0.02	0.690 ± 0.03	0.872 ± 0.02	0.120 ± 0.01	0.889 ± 0.03	0.710 ± 0.02	0.848 ± 0.03	0.128 ± 0.02
w/o Cross-Modal Attention	0.835 ± 0.03	0.665 ± 0.02	0.880 ± 0.02	0.114 ± 0.02	0.869 ± 0.02	0.690 ± 0.03	0.856 ± 0.02	0.123 ± 0.02
w/o Adaptive Feature Selection	0.842 ± 0.02	0.678 ± 0.03	0.876 ± 0.03	0.116 ± 0.02	0.876 ± 0.03	0.701 ± 0.02	0.853 ± 0.02	0.125 ± 0.02
Ours	0.815 ± 0.02	0.635 ± 0.03	0.892 ± 0.02	0.108 ± 0.01	0.850 ± 0.03	0.659 ± 0.02	0.864 ± 0.03	0.116 ± 0.02
p-value vs. w/o Feature Extraction	0.002	0.003	0.002	0.004	0.005	0.006	0.008	0.007

**TABLE 5:** Performance Benchmarking of our approach against leading techniques on our model across OCT and LIDC-IDRI datasets.

Model variant	OCT dataset				LIDC-IDRI dataset			
	RMSE ↓	MAE ↓	R-Squared ↑	MAPE ↓	RMSE ↓	MAE ↓	R-Squared ↑	MAPE ↓
w/o Feature Extraction	1.210 ± 0.03	0.960 ± 0.02	0.799 ± 0.02	0.169 ± 0.02	1.282 ± 0.02	1.014 ± 0.03	0.776 ± 0.02	0.177 ± 0.02
w/o Cross-Modal Attention	1.195 ± 0.02	0.945 ± 0.03	0.807 ± 0.03	0.165 ± 0.01	1.268 ± 0.03	1.000 ± 0.02	0.782 ± 0.02	0.174 ± 0.02
w/o Adaptive Feature Selection	1.202 ± 0.03	0.950 ± 0.02	0.804 ± 0.02	0.167 ± 0.02	1.275 ± 0.02	1.007 ± 0.03	0.779 ± 0.02	0.175 ± 0.02
Ours	1.160 ± 0.03	0.905 ± 0.02	0.828 ± 0.02	0.156 ± 0.01	1.233 ± 0.02	0.965 ± 0.03	0.803 ± 0.02	0.164 ± 0.02
p-value vs. w/o Feature Extraction	0.003	0.004	0.003	0.005	0.006	0.006	0.009	0.008

The removal of Feature Extraction leads to a significant increase in RMSE and MAE, indicating that it is critical for capturing complex patterns in textual and behavioral data. Excluding Cross-Modal Attention results in a moderate performance drop, particularly in R-Squared, which suggests that it plays an essential role in improving model generalization. The removal of Adaptive Feature Selection primarily affects MAPE, reinforcing its importance in reducing relative prediction errors. As in the previous datasets, the full model consistently achieves the best performance across all evaluation metrics, highlighting the synergistic effect of its components. The ablation study confirms that each component of our model contributes to its overall effectiveness. Feature Extraction enhances representation learning, Cross-Modal Attention improves model robustness, and Adaptive Feature Selection refines prediction accuracy. The superior performance of our complete model across all datasets demonstrates that our proposed architecture is well-structured and effectively leverages multiple features for recommendation tasks. These findings validate the necessity of our design choices and reinforce the advantages of our approach over alternative methods.

The observed performance degradation in the ablation studies can be explained by examining the unique role and interactions of each component. Removing Cross-Modal Attention causes RMSE to increase from 0.815 to 0.835 on PhysioNet and from 1.233 to 1.268 on LIDC-IDRI. This highlights that without attention, the model cannot dynamically capture context-aware relevance among modalities, leading to inefficient fusion of modality-specific information. The Adaptive Feature Selection module, when removed, causes similar degradation, indicating its importance in filtering out redundant or noisy modality features. Without this mechanism, irrelevant modality channels may dominate, impairing model robustness. Most notably, we observe compounded degradation when either of the above modules is removed alongside the Graph Representation Learning (GCN). This suggests that attention and GCN act synergistically: attention allows the model to discover fine-grained modality relevance, while the GCN structurally propagates these refined signals across a learned graph, modeling global inter-modal dependencies. In isolation, attention mechanisms treat interactions independently, and GCN lacks soft alignment cues. Their interaction is therefore critical—attention strengthens node semantics, while GCN leverages inter-node structure for deeper reasoning. The presence of both leads to stronger feature representations and improved generalization, as evidenced by the consistent superiority of the full model across all metrics and datasets.

To clarify the unique contributions of AMFN, we conducted comparative experiments using a series of established temporal and attention-based models, including Transformer (Cross-Attention), TFT, N-BEATS, and TCN. As shown in Table 6, our AMFN achieves the best performance across all metrics on the PhysioNet dataset. AMFN improves RMSE from 0.863 (Transformer) to 0.815, and R-Squared from 0.861 to 0.892, reflecting a substantial gain in both prediction accuracy and variance explanation. These improvements are driven by AMFN’s integration of dynamic attention, temporal encoding, and graph-based reasoning. While Transformer and TFT use attention to capture sequential relevance, they do not explicitly model structural modality dependencies. N-BEATS and TCN improve temporal modeling but treat modalities independently, without learning inter-modality relationships. In contrast, AMFN builds a modality graph based on learned temporal similarities, enabling the model to propagate refined, semantically aligned signals between modalities. Moreover, AMFN’s adaptive feature selection allows it to filter out modality-specific noise, preserving only the most informative features in each context. This is particularly useful for clinical settings where signal quality varies across modalities. The use of context-aware fusion ensures that the model dynamically adjusts modality contributions based on the specific task scenario—something fixed-weight fusion strategies cannot achieve. Collectively, these components contribute to a more expressive and robust multimodal learning process, which is both statistically superior and clinically interpretable. This hybrid architecture, combining cross-modal attention and structured reasoning, distinguishes AMFN from purely sequential or attention-only approaches and explains its consistent empirical advantage in modeling complex biomedical time series.

**TABLE 6:** Comparative Performance between Existing Cross-Attention and Temporal Models vs Our AMFN on the PhysioNet Dataset.

Model	RMSE ↓	MAE ↓	R-Squared ↑	MAPE ↓
Transformer (Cross-Attention) Apruzzi (2022)	0.863 ± 0.02	0.678 ± 0.02	0.861 ± 0.03	0.120 ± 0.01
TFT Januschowski et al. (2022)	0.854 ± 0.03	0.670 ± 0.02	0.867 ± 0.02	0.118 ± 0.02
N-BEATS Wang et al. (2022)	0.848 ± 0.02	0.665 ± 0.02	0.871 ± 0.02	0.116 ± 0.02
TCN Fan et al. (2023)	0.839 ± 0.03	0.658 ± 0.02	0.875 ± 0.02	0.113 ± 0.01
AMFN (Ours)	0.815 ± 0.02	0.635 ± 0.03	0.892 ± 0.02	0.108 ± 0.01

To further investigate the clinical applicability of our model across diverse biomedical tasks, we evaluated whether minimal task-specific adaptations could improve predictive performance relative to the original unified pipeline. We selected two datasets with distinct task types: PhysioNet for binary outcome prediction using physiological time series, and OCT for image-based retinal disease classification. While the unified model is designed to generalize across modalities, it does not incorporate mechanisms that account for the statistical characteristics of different task domains. We hypothesized that tailoring the architecture or loss function at minimal cost could improve task alignment and model utility. On the PhysioNet dataset, we modified the classification head to include a task-specific dense projection layer and replaced the standard cross-entropy loss with a focal loss, which better handles class imbalance often present in mortality prediction. On the OCT dataset, where image granularity and multi-scale retinal structures are critical, we replaced the final transformer-based decoder with a multi-scale convolutional decoder that emphasizes spatial context. The results are summarized in Table 7. For PhysioNet, the adapted model achieved an increase in accuracy from 85.3% to 87.1% and an F1-score improvement from 0.873 to 0.890. For OCT, performance improved from 82.4% to 85.0% in accuracy and from 0.843 to 0.865 in F1-score. These consistent gains demonstrate that even lightweight, task-aware modifications to the base framework can significantly enhance predictive accuracy. Importantly, these modifications do not require changes to the multimodal backbone or fusion strategy, preserving the generality of the core design while improving domain relevance. This supports the claim that our framework is flexible and can be extended to accommodate varying biomedical objectives through modular adaptation.

**TABLE 7:** Performance comparison between unified pipeline and task-specific adaptation.

Dataset	Task type	Model variant	Accuracy (%)	F1-score
PhysioNet	Outcome Prediction	Unified Pipeline	85.3	0.873
PhysioNet	Outcome Prediction	Task-Specific Classifier Head + Focal Loss	87.1	0.890
OCT	Retinal Disease Classification	Unified Pipeline	82.4	0.843
OCT	Retinal Disease Classification	Task-Specific Multi-Scale CNN Decoder	85.0	0.865

To further validate our model’s performance claims, we conducted additional experiments comparing AMFN with recent large-scale pretrained biomedical models, namely, BioGPT and MedFormer. These models have demonstrated strong results in various medical natural language and multimodal tasks due to their scale and extensive pretraining. As shown in Table 8, while both BioGPT and MedFormer outperform traditional deep learning baselines, AMFN consistently achieves the best results across all metrics on the PhysioNet dataset. AMFN reduces RMSE from 0.848 (BioGPT) and 0.832 (MedFormer) to 0.815, and improves R-Squared from 0.878 to 0.892. This improvement reflects AMFN’s ability to tailor its representation learning to time-series signal characteristics rather than relying on general-purpose textual embeddings or static fusion strategies. The superiority of AMFN stems from its integration of domain-specific inductive biases, including temporal alignment, adaptive feature selection, and graph-structured cross-modal reasoning. Unlike pretrained models optimized for language or vision-language tasks, AMFN is designed to natively process multimodal physiological signals and account for their temporal dependencies and cross-modality structure, which makes it more suitable for real-time biomedical monitoring scenarios.

**TABLE 8:** Performance comparison of AMFN with large-scale pretrained biomedical models on PhysioNet dataset.

Model	RMSE ↓	MAE ↓	R-Squared ↑	MAPE ↓
BioGPT Luo et al. (2022)	0.848 ± 0.02	0.660 ± 0.03	0.871 ± 0.03	0.117 ± 0.02
MedFormer Wang et al. (2024)	0.832 ± 0.03	0.652 ± 0.02	0.878 ± 0.02	0.113 ± 0.02
AMFN (Ours)	0.815 ± 0.02	0.635 ± 0.03	0.892 ± 0.02	0.108 ± 0.01

## 5 Discussion

Model interpretability is a critical aspect for the clinical applicability of AI systems, as healthcare professionals require not only accurate but also transparent predictions. Our framework reflects interpretability at multiple architectural levels, which is further validated by empirical results. The cross-modal attention mechanism allows dynamic weighting of each modality based on its contextual relevance. These attention weights are extractable and can be visualized to reveal modality-level contributions. For example, in PhysioNet experiments, physiological signals such as heart rate variability and blood pressure often received higher attention scores during adverse event predictions, reflecting their clinical importance. The adaptive feature selection module applies feature-level weighting within each modality, identifying the most informative features while suppressing irrelevant noise. The quantitative impact of this mechanism is evident in our ablation studies: removing adaptive feature selection increases RMSE from 0.815 to 0.842 and decreases R-Squared from 0.892 to 0.876 on the PhysioNet dataset; similarly, RMSE rises from 1.233 to 1.275 and MAPE from 0.164 to 0.175 on the LIDC-IDRI dataset. These performance drops confirm the module’s role in refining feature relevance, which inherently supports interpretability. Graph-based representation learning captures explicit inter-modal dependencies through the learned adjacency matrix. Visualizing this graph structure allows clinicians to understand how different modalities interact and jointly influence the model’s predictions. Collectively, these design elements ensure that the model not only achieves superior accuracy but also maintains transparency regarding how specific data sources contribute to each decision, thereby supporting clinical trust and usability.

Beyond improvements in predictive accuracy, the proposed model is designed with real-world clinical integration in mind, particularly in the context of perioperative monitoring and ICU-based decision support. In surgical care, multimodal data such as ECG waveforms, oxygen saturation, lab values, and operative notes are collected in real time. Our architecture enables clinicians to continuously predict patient deterioration risk by fusing this heterogeneous data into a unified and interpretable prediction signal. For instance, in early postoperative periods, the model can prioritize vital signs and physiological signals over text-based EHR notes to generate rapid alerts when cardiac or respiratory anomalies are detected. The attention mechanisms and adaptive weighting introduced in our model provide a degree of interpretability that aligns with clinical reasoning. By examining modality-specific attention weights and cross-modal alignment scores, clinicians can identify which data sources are driving the model’s prediction. This enables actionable insights rather than black-box recommendations. Our architecture also supports uncertainty-aware prediction, allowing for confidence estimation, which is critical in triaging patients based on the reliability of model outputs. For deployment, the modularity of our system supports integration into existing hospital IT pipelines. Each module can be containerized and deployed independently on edge or cloud infrastructure, depending on latency and resource requirements. The model has been trained and validated using real-world datasets that mirror clinical heterogeneity, which supports its generalizability. While further validation in prospective settings is warranted, the current design offers a viable path toward implementation in real-time clinical monitoring platforms and decision support systems.

## 6 Conclusions and future work

In this study, we explored the potential of multimodal deep learning in biomedical time series prediction, addressing the limitations of unimodal learning approaches that fail to fully utilize heterogeneous data sources. Our proposed framework, the Adaptive multimodal Fusion Network (AMFN), effectively captures inter-modal dependencies through attention-based alignment, graph-based representation learning, and modality-adaptive fusion. The Dynamic Cross-Modal Learning Strategy (DCMLS) is introduced to optimize feature selection, mitigate modality-specific noise, and incorporate uncertainty-aware learning, thereby improving model generalization. Experimental evaluations on biomedical datasets demonstrate that our approach surpasses existing methods in predictive accuracy, robustness, and interpretability. By bridging the gap between different biomedical data modalities—such as physiological signals, imaging, and electronic health records—our framework contributes to more reliable AI-driven disease diagnosis and treatment planning.

Despite its promising results, the proposed framework presents two main limitations. Modality misalignment and data heterogeneity remain challenges, especially when dealing with highly variable patient data from different sources. While our attention-based alignment strategy improves information integration, further enhancements—such as self-supervised learning or domain adaptation techniques—could further refine the robustness of multimodal fusion. Computational complexity is a concern, as our model incorporates multiple processing layers, including graph-based representations and adaptive fusion mechanisms. This may hinder real-time deployment in clinical settings where rapid decision-making is critical.

Another important aspect that warrants further discussion is the current reliance on predefined modality pairs during the cross-modal alignment phase. While our approach utilizes positive modality pairs with shared semantic meaning to compute the alignment loss, this design may introduce certain constraints when applied to novel or highly heterogeneous datasets where clear pairwise modality relationships are either unknown or inconsistent. In real-world biomedical scenarios, new data sources may contain unstructured or partially missing modalities, making it challenging to accurately define such modality pairs beforehand. This rigidity could potentially limit the model’s adaptability and flexibility when integrating unforeseen data types or when dealing with incomplete patient records. To address this limitation, future research directions could explore more flexible alignment mechanisms such as unsupervised or semi-supervised cross-modal matching, where latent representations are dynamically aligned based on shared contextual information rather than strict pairwise definitions. Moreover, incorporating contrastive learning frameworks that leverage self-supervised objectives could allow the model to autonomously discover latent cross-modal correspondences from the available data distribution. Another promising direction involves the application of domain adaptation techniques to adjust the alignment strategy when transferring the model across different clinical environments with varying modality compositions. These enhancements would further improve the scalability and robustness of the model in broader biomedical applications while minimizing the dependency on predefined modality pairings.

## Data Availability

The original contributions presented in the study are included in the article/supplementary material, further inquiries can be directed to the corresponding author.
